# Glycyrrhizic Acid Suppresses the Development of Precancerous Lesions via Regulating the Hyperproliferation, Inflammation, Angiogenesis and Apoptosis in the Colon of Wistar Rats

**DOI:** 10.1371/journal.pone.0056020

**Published:** 2013-02-14

**Authors:** Rehan Khan, Abdul Quaiyoom Khan, Abdul Lateef, Muneeb U. Rehman, Mir Tahir, Farrah Ali, Oday O. Hamiza, Sarwat Sultana

**Affiliations:** Section of Molecular Carcinogenesis and Chemoprevention, Department of Medical Elementology and Toxicology, Jamia Hamdard (Hamdard University), Hamdard Nagar, New Delhi, India; Health Canada, Canada

## Abstract

**Background:**

Colon carcinogenesis is a multistep process and it emanates from a series of molecular and histopathological alterations. Glycyrrhizic acid (GA) is a natural and major pentacyclic triterpenoid glycoside of licorice roots extracts. It has several pharmacological and biological properties such as anti-inflammatory, anti-viral, and anti-cancer. In the present study, we investigated the chemopreventive potential of GA against 1,2-dimethyhydrazine (DMH)-induced precancerous lesions i.e., aberrant crypt foci (ACF) and mucin depleted foci (MDF), and its role in regulating the hyperproliferation, inflammation, angiogenesis and apoptosis in the colon of Wistar rats.

**Methods:**

Animals were divided into 5 groups. In group III, IV and V, GA was administered at the dose of 15 mg/kg b. wt. orally while in group II, III and IV, DMH was administered subcutaneously in the groin at the dose of 20 mg/kg b.wt once a week for first 5 weeks and animals were euthanized after 9 weeks.

**Results:**

GA supplementation suppressed the development of precancerous lesions and it also reduced the infiltration of mast cells, suppressed the immunostaining of Ki-67, NF-kB-p65, COX-2, iNOS and VEGF while enhanced the immunostaining of p53, connexin-43, caspase-9 and cleaved caspase-3. GA treatment significantly attenuated the level of TNF-α and it also reduced the depletion of the mucous layer as well as attenuated the shifting of sialomucin to sulphomucin.

**Conclusion:**

Our findings suggest that GA has strong chemopreventive potential against DMH-induced colon carcinogenesis but further studies are warranted to elucidate the precise mechanism of action of GA.

## Introduction

Colon cancer is one of the most common, best-understood neoplasms from a genetic point of view, yet it remains the leading cause of cancer-related mortality in men and women [1], [2]. Colon carcinogenesis is a multistep process and it emanate from a series of molecular and histopathological alterations [3]. It thought to arise by the accretion of genetic alterations involving a variety of oncogenes and tumor suppressor genes that transform normal colonic epithelium into an invasive carcinoma, with aberrant crypt foci (ACF) and mucin depleted foci (MDF) as putative preneoplastic lesions in this transformation process [3]–[5]. ACF were first discovered in the colon of carcinogen treated rodent [6] and they have also been observed in patients with sporadic colorectal cancer (CRC) and with familial adenomatous polyposis (FAP) [7]–[9]. They are identified by their elevated crypts, thicker colonic epithelial cell lining and increased pericryptal zone comparative to normal crypts [6]. They exhibit preneoplastic features e.g., dysplasia [10]–[12], hyperproliferation [6], [13], [14], K-ras mutations [15]–[18], and over-expression of c-fos [19], β-catenin [10], [20] and cyclin D1 [10]. MDF were first discovered in the colon of carcinogen treated rats [21] and they have also been observed in patients with FAP and with sporadic CRC [22], [23]. MDF, devoid of mucin which is secreted by goblet cells, also exhibit preneoplastic features e.g., dysplasia [21], [23]–[25], mutations in β-catenin [26] and Apc [24] gene, over-expression of survivin [27], cyclooxygenase-2 (COX-2), inducible nitric oxide synthase (i-NOS) and macrophages [28] and reduced expression of MUC2 (a mucin abundantly expressed in the normal colon) and intestinal trefoil factor, a marker of goblet cell lineage [25]. While the expression of p21 and p16 (inbibitors of cyclin-dependent kinases) have been found to be reduced in ACF as well as MDF [29]. On the basis of above evidences, both ACF and MDF are considered to be putative early biomarkers of colon cancer.

Among the panoply of inflammatory mediators, nuclear factor kappaB (NF-kB) and tumor necrosis factor-α (TNF-α) are the key factors involved in cancer-related inflammation [30]. In inflammatory cells as well as in cells at risk of transformation by carcinogens, NF-κB mediates the transactivation of genes encoding inflammatory cytokines (e.g., TNF-α), anti-apoptotic factors (e.g., *BCL-2),* cyclooxygenase-2 (COX2), inducible nitric oxide synthase (iNOS) and angiogenic factors (e.g., *VEGF),* [30], [31]. Mast cells play an important role in the initiation of inflammation. Mast cells have also been reported as being an essential hematopoietic component of the development of adenomatous polyps [32]. Increased mast cell numbers have also been observed in patients with ulcerative colitis and Crohn’s disease, both of which are risk factors in colon cancer susceptibility [33], [34]. In a 1,2-dimethylhydrazine–induced intestinal tumor model, the incidence of intestinal cancer was significantly reduced in mast cell–deficient KitW/KitW-v mice [35]. Although the mechanisms by which mast cells contribute to carcinogenesis are not understood. Mast cells are the only tissue-resident cells with granules containing preformed tumor necrosis factor-α (TNF-α), and releasing this cytokine from mast cells is important for the initiation of an inflammatory response [36]. TNF-α is a pro-inflammatory cytokine that creates tumor microenvironment fostering tumor development by induction of tumor promoting cytokines, release of matrix metalloproteinases (MMPs) and pro-angiogenic activity [37]. Recently, it has been reported that the expression of inflammatory markers e.g., COX-2, and i-NOS are also enhanced together with orchestration of intracolonic infiltration of macrophage in MDF similarly as in colonic tumors. This might be due to reduced expression of MUC2 in MDF which consequently leads to the focal loss of the protective mucous layer. Thus, MDF are more exposed to toxicant present in the colon which ultimately results in the activation of inflammation in MDF. It has also been reported that inflammation provides a nidus for the development of MDF and colonic tumors, further supporting the concept that MDF are early preneoplastic lesions [28].

There is unequivocal evidence that chemoprevention is a pragmatic approach to inhibit or suppress the colon carcinogenesis or the development of ACF and MDF into adenoma or adenocarcinoma. ACF and MDF are being exploited as short-term bioassays to assess the chemopreventive potential of natural products against colon carcinogenesis [38]. Thus, inhibiting or suppressing the development of ACF and MDF by natural products may be able to dampen the subsequent progression to colon cancer.

Glycyrrhizic acid or glycyrrhizin is a natural and major pentacyclic triterpenoid glycoside of licorice roots extracts [39]. It has several pharmacological and biological properties such as anti-inflammatory, anti-viral [40], [41] and anti-cancer [42]–[44]. After oral administration in humans as well as in rats glycyrrhizic acid is metabolised in the gastrointestinal tract by glucuronidases into glycyrrhetic acid, its biologically active metabolite. Glycyrrhetic acid is completely absorbed [45]. In humans no glycyrrhizic acid was detected in plasma following an oral dose of 100–800 mg. In rats glycyrrhizic acid plasma levels were only detectable after high oral doses of 50–500 mg/kg, demonstrating low oral bioavailability (4% after 200 mg/kg) [46]. It also induces apoptosis in several cancer cell lines such as human hepatoma (HLE), promyelotic leukemia (HL-60) and stomach cancer (KATO III) and prostate cancer cell lines (DU-145 and LNCaP) [47]–[49]. Glycyrrhizic acid is commonly used in Japan as a therapeutic agent for the control and treatment of chronic viral hepatitis [50]–[52].

In the light of above facts, we hypothesized that glycyrrhizic acid may have chemopreventive potential against DMH-induced ACF and MDF and its role in regulating the hyperproliferation, inflammation, angiogenesis and apoptosis in the colon of Wistar rats.

## Materials and Methods

### Chemicals

Poly-L-lysine, tris-base, tris-HCl, bovine serum albumin (BSA), Mayer’s hematoxylin, alcian blue 8GX, neutral red, toluidine blue, propidium iodide, methylene blue, N,N’-dimethyl-p-phenylene diamine, N,N’-dimethyl-m-phenylene diamine, 1,2-dimethylhydrazine (DMH), glycyrrhizic acid were obtained from Sigma (Sigma Chemical Co., St Louis, MO). Poly-HRP plus ONE detection System (Thermo Scientific). Hydrogen peroxide, xylene, ethanol, tween-20, sodium potassium tartrate, di-sodium hydrogen phosphate, sodium dihydrogen phosphate, sodium citrate, acetone, methanol, formaldehyde, acetic acid, hydrochloric acid (HCl) and sodium hydroxide were purchased from E. Merck Limited, India.

### Ethics Statement

All procedures for using experimental animals were checked and permitted by the “Institutional Animal Ethical Committee (IAEC)” that is fully accredited by the Committee for Purpose of Control and Supervision on Experiments on Animals (CPCSEA) Chennai, India. The animals were provided by “Central Animal House Facility, Jamia Hamdard, whose registration number and date of registration are 173/CPCSEA and 28^th^ Jan 2000. Approval ID/project number for this study is 780.

### Animals

Four to six-weeks-old, male albino rats (120–150 g) of Wistar strain were obtained from Central Animal House of Hamdard University, New Delhi, India. All procedures for using experimental animals were checked and permitted by the “Institutional Animal Ethical Committee (IAEC)” that is fully accredited by the Committee for Purpose of Control and Supervision on Experiments on Animals (CPCSEA) Chennai, India. Approval ID/project number for this study is 740. They were housed in polypropylene cages in groups of four rats per cage and were kept in a room maintained at 25±2°C with a 12 h light/dark cycle. They were allowed to acclimatize for one week before the experiments and were given free access to standard laboratory animal diet and water ad libitum.

### Preparation of Carcinogen

DMH was weighed and dissolved in distilled water containing 1 mM EDTA to ensure the stability of the chemical just prior to use and the pH was adjusted to 6.5 with 1 M NaOH solution.

### Treatment Regimen

To study the effect of treatment with glycyrrhizic acid against DMH-induced ACF and MDF in colon, 40 male Wistar rats were randomly allocated to 5 groups of 8 rats each.

#### Group 1 (Control)

Rats received basal diet along with distilled water (5 ml/kg b.wt.).

#### Group 2 (DMH)

Rats were administered with subcutaneous injection of DMH at the dose of 20 mg/kg b.wt. once a week for first 5 weeks.

#### Group 3 (DMH+GA) (I)

Rats were administered with DMH as in group 2 and also fed glycyrrhizic acid (15 mg/kg b.wt. orally) every day for the first 5 weeks starting 1 week before carcinogen treatment (Initiation- I).

#### Group 4 (DMH+GA) (PI)

Rats were administered with DMH as in group 2 and also fed glycyrrhizic acid (15 mg/kg b.wt. orally) 2 days after the last injection of the carcinogen and continued till the end of the experiment (Post-initiation-PI).

#### Group 5 (Only GA)

**Figure 1:**
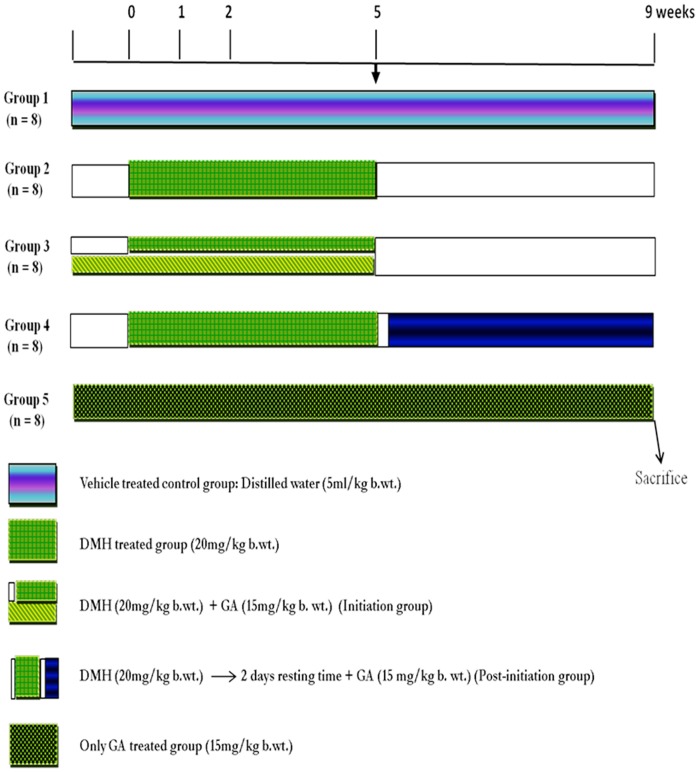
Schematic representation of the experimental design.

Rats received basal diet**+**glycyrrhizic acid (15 mg/kg b.wt. orally) dissolved in distilled water everyday throughout the experiment. (Figure 1).

All the rats were anaesthetized with mild anesthesia and sacrificed by cervical dislocation after 9 weeks.

### Immunohistochemical Staining for Detection of Ki-67, VEGF, i-NOS, COX-2, NF-kB-p65, p53, Caspase-9 and Cleaved Caspase-3

Immunohistochemistry was done as described by Khan et al. 2012 [53]. Primary antibodies: Anti-rat Ki-67 epitope specific rabbit antibody (dilution 1∶300, Neomarkers, Fremont CA); Anti-rat NF-kB-p65 rabbit antibody (dilution 1∶100, Biolegend); Anti-rat COX-2 polyclonal antibody (dilution 1∶300, Santa Cruz Biotechnology, Inc.); Anti-rat i-NOS rabbit antibody (dilution 1∶100) Neomarkers, Fremont CA); Anti-rat VEGF rabbit polyclonal antibody (dilution 1∶300, Neomarkers, Fremont CA); Anti-rat p53 rabbit polyclonal antibody (dilution1∶100, Santa Cruz); Anti-rat caspase-9 rabbit polyclonal antibody (dilution 1∶300, Neomarkers, Fremont CA); Anti-rat cleaved caspase-3 rabbit monoclonal antibody (dilution 1∶400, Cell Signalling).

### Fluorescent Immunohistochemical Staining for Detection of Connexin-43

Thick sections of 4 µm were cut from formalin-fixed, paraffin-embedded tissue blocks and mounted on poly-l-lysine coated microscopic slides. Paraffinized sections were dewaxed in xylene and rehydrated through graded series of ethanol to water followed by antigen retrieval in sodium citrate buffer (10 mM, pH 6.0). The slides were then allowed to cool for 15 minutes and washed 3 times with tris-buffered saline (TBS) for 5 minutes each. The slides then subject to power block (UltraVision Plus Detection System, Thermo Scientific) for 10 min to block non-specific binding. After rinsing the sections in TBS, the slides were incubated overnight at 4°C with primary antibody inside humidified chamber and then were washed in TBS. The sections were incubated with FITC-conjugated secondary antibody (dilution 1∶50) for 20 min and then were rinsed in TBS. The sections were counterstained with propidium iodide to visualize nuclei, mounted by using mounting media and then observed under the light microscope (Olympus BX51). Fluorescein (FITC)-conjugated AffiniPure Goat Anti-Rat secondary antibody (dilution 1∶50, Jackson ImmunoResearch Lab Inc.). Primary antibody of anti-rat connexin-43 mouse monoclonal antibody (dilution 1∶400, Santa Cruz).

### Mast Cell Staining

For detection of mast cells, colon was fixed in methacarn solution (methanol: chloroform: glacial acetic acid:: 4∶2:1) overnight at 4°C and then fixed in 4% neutral buffered formalin for additional 24 hours. The colonic sections of 4 µm were cut from formalin-fixed, paraffin-embedded tissue blocks and mounted on poly-l-lysine coated microscopic slides. Paraffinized sections were dewaxed in xylene and rehydrated through graded series of ethanol to water. The sections were stained with 0.1% toluidine blue (pH 2.3) in 1% sodium chloride solution for 5 min. The slides were then washed three times in distilled water and dehydrated quickly in alcohol, clear in xylene and mounted by using mounting media. The slides were then evaluated under the light microscope (Olympus BX51). Staining with toluidine blue permits the identification of mast cells because mast cell granules stain metachromatically, resulting in deep purplish-blue granular cytoplasmic staining.

### Assay for Tumor Necrosis Factor Alpha (TNF-α)

TNF-α levels were determined by rat TNF-α kit (eBioscience, Inc., San Diego., USA). The method is based on enzyme-linked immuno-sorbent assay (ELISA). We have performed measurement of TNF-α in the colonic tissue by ELISA. Samples were prepared in phosphate buffered saline (PBS) containing protease inhibitor cocktail. Analysis was performed by Elisa Plate Reader (Benchmark plus, BioRad) according to the manufacturer’s instruction.

### Aberrant Crypt Foci (ACF) Assay

ACF assay was done by the method of Bird, 1987 [6]. Colons picked up in random order were stained for 6 min in a 0.05% filtered solution of methylene blue. The numbers of ACF per colon were counted under light microscope (Olympus BX51) at x40 magnification.

### Mucin Depleted Foci (MDF) Assay

MDF assay was done by the method of Caderni et al., 2003 [21]. Colons, after being scored for ACF, were stained with high iron diamine-Alcian blue (HID-AB) procedure to evaluate mucin production. The numbers of MDF per colon were counted under light microscope (Olympus BX51) at x40 magnification.

### High iron Diamine-alcian blue (HID-AB) Staining for Sulphomucin and Sialomucin

The colonic sections of 4 µm were cut from formalin-fixed, paraffin-embedded tissue blocks and mounted on poly-l-lysine coated microscopic slides. Paraffinized sections were dewaxed in xylene and rehydrated through graded series of ethanol to water. The colonic sections were stained with high iron diamine (HID) solution for 18 hours at room temperature in the dark and washed 3 times with distilled water. Stains the sections with 1% alcian blue (dissolved in 3% acetic acid solution) for 30 min and washed 3 times with 80% ethanol. Then washed with distilled water and dehydrated quickly in alcohol, clear in xylene and mounted by using mounting media. The slides were then evaluated under the light microscope (Olympus BX51).

### Alcian Blue-neutral red (AB-NR) Staining for Mucin Analysis

The colonic sections of 4 µm were cut from formalin-fixed, paraffin-embedded tissue blocks and mounted on poly-l-lysine coated microscopic slides. Paraffinized sections were dewaxed in xylene and rehydrated through graded series of ethanol to water. The sections were stained with 1% Alcian blue (pH 2.5) in 3% acetic acid solution for 30 min and then rinsed for 1 min in 3% acetic acid solution to prevent non-specific staining. The slides were then washed in distilled water and the sections were then counterstained with neutral red (0.5% aqueous solution) for 20 sec, dehydrated in alcohol and mounted by using mounting media. The slides were then evaluated under the light microscope (Olympus BX51).

### Histology

Histology was done as described by Khan et al. 2011 [4]. These colonic sections that were stained with haematoxylin and eosin (H&E) were observed under light microscope (Olympus BX51) at 10x and 40x magnifications to investigate the histoarchitecture of colonic mucosa.

### Statistical Analysis

The data from individual groups were presented as the mean ± SD. Differences between groups were analyzed using analysis of variance (ANOVA) followed by Tukey-Kramer multiple comparisons test and minimum criterion for statistical significance was set at p<0.05 for all comparisons.

## Results

### Effect of Glycyrrhizic Acid and DMH on the Development of ACF in Colonic Tissue

**Figure 2:**
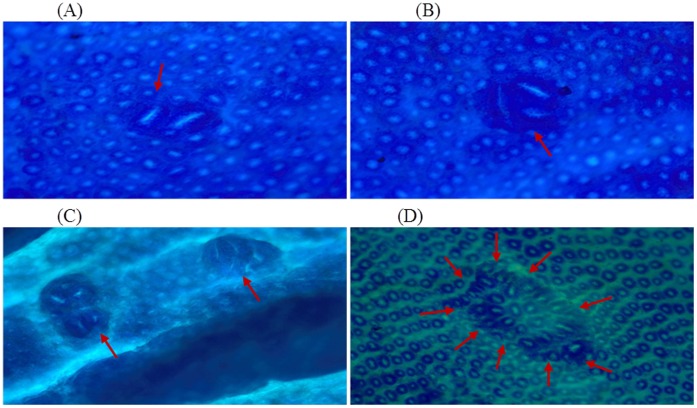
Topographical view of ACF. The rat colon showing round and elongated ACF with different crypt multiplicities. The colons were opened, stained with methylene blue and observed on a glass slide. Original magnification: 10x.

**Figure 3:**
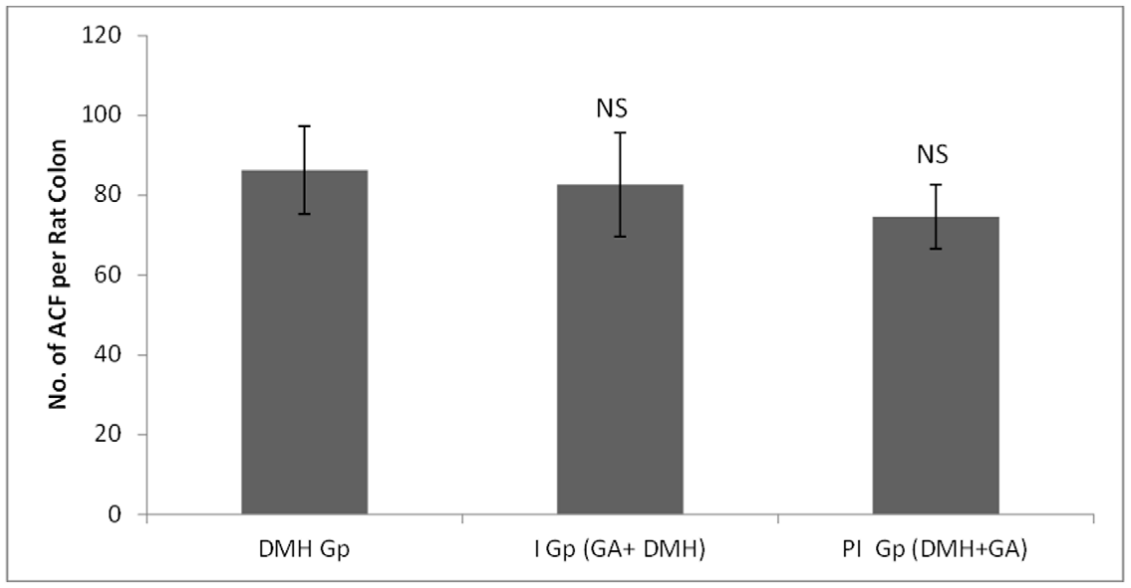
Effects of glycyrrhizic acid and DMH on incidence of ACF per rat colon. Values are expressed as Mean ± SD. Glycyrrhizic acid non-significantly suppressed the development of ACF in Group 3 and Group 4 as compared to DMH treated group (Group 2). NS- Non-significant.

In DMH treated group (Group II), the number of ACF/colon is 86.33±11 while supplementation with glycyrrhizic acid in Group III (82.67±13) and Group IV (74.67±8) were non-significantly reduced the number of ACF. Original magnification: 10x. (Figure 2 & 3).

### Effect of Glycyrrhizic Acid and DMH on the Development of MDF in Colonic Tissue

**Figure 4:**
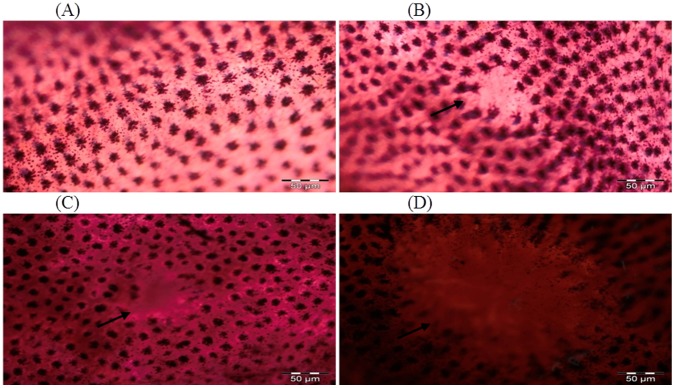
Topographical view of MDF. MDF in the rat colon showing depleted mucin as indicated by arrow. The colons were opened, stained with high iron diamine (HID) and alcian blue (AB). Original magnification: 10x.

**Figure 5:**
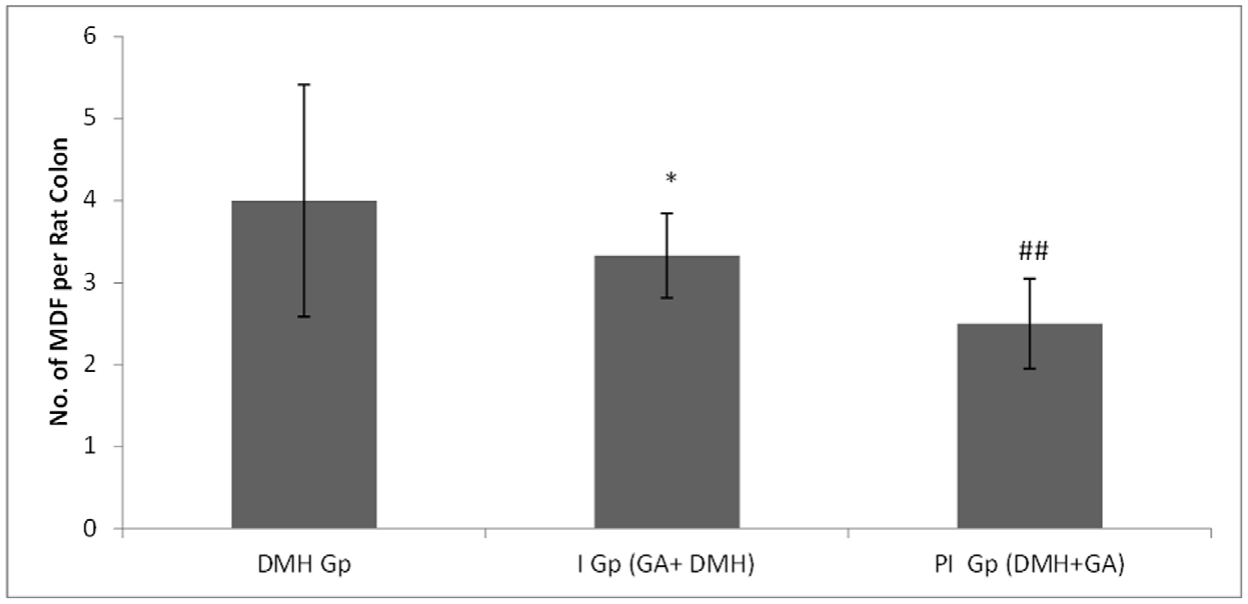
Effects of DMH and glycyrrhizic acid on incidence of MDF per rat colon. Values are expressed as Mean ± SD. Glycyrrhizic acid significantly suppressed the development of MDF in Group 3 and Group 4 as compared to DMH treated group (Group 2).

In DMH treated group (Group II), the number of MDF/colon is 4±1.317 while supplementation with glycyrrhizic acid significantly (*p<0.05) reduced the number of MDF in Group III (2.83±0.516) as compared to Group II. In Group IV, treatment with glycyrrhizic acid significantly (##p<0.01) reduced the number of MDF (2.27±0.547) as compared to Group II. Original magnification: 10x. (Figure 4 & 5).

### Effect of Glycyrrhizic Acid and DMH on the Mucin Staining in Colonic Tissue

**Figure 6:**
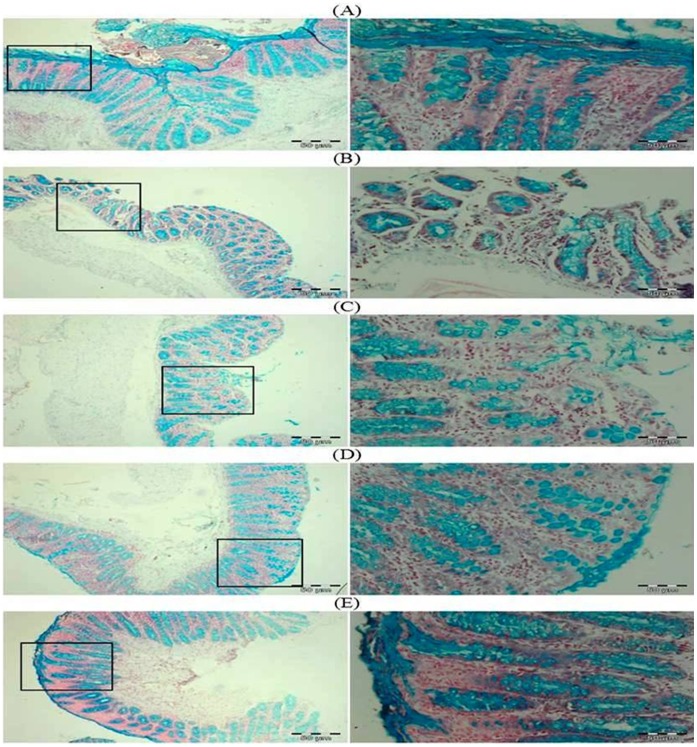
Photomicrographs depicting mucin staining. In DMH treated group (Group II), there is regional depletion of mucin in the form of mucous layer (blue in color). Treatment with glycyrrhizic acid decreased the depletion of the mucous layer in Group IV as compared to Group II. No effects of glycyrrhizic acid on mucous layer in Group III as compared to Group II. There is no depletion of the mucous layer in colonic sections of Group I and Group V. Insets at the right panel show a magnified view (40x magnifications) of the insets showed at the left panel (10x magnifications).

In DMH treated group (Group II), there is regional depletion of mucous layer (blue in color). Treatment with glycyrrhizic acid attenuated the depletion of the mucous layer in Group IV but not in Group III as compared to Group II. There is no depletion of the mucous layer in colonic sections of Group I and Group V. (Figure 6).

### Effect of Glycyrrhizic Acid and DMH on the Colonic Sulphomucin and Sialomucin

**Figure 7:**
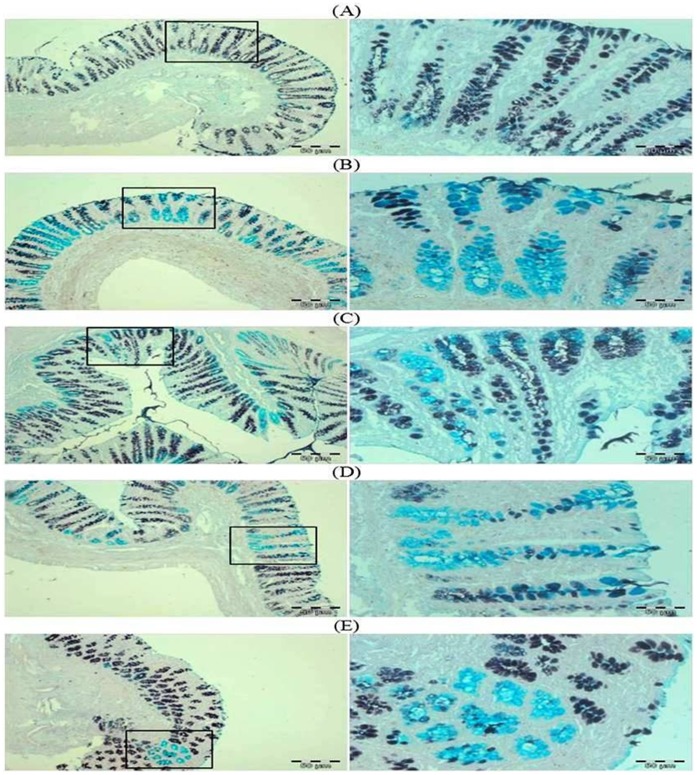
Photomicrographs depicting sulphomucin and sialomucin staining. In DMH treated group, predominance of sialomucin (blue color) or shift from sulphomucin (brown color) to sialomucin (blue color) was observed. While treatment with glycyrrhzic acid markedly attenuated this shifting or there is predominance of sulphomucin as in control group. Insets at the right panel show a magnified view (40x magnifications) of the insets showed at the left panel (10x magnifications).

In DMH treated group, predominance of sialomucin (blue color) or shift from sulphomucin (brown color) to sialomucin (blue color) was observed. While treatment with glycyrrhzic acid markedly attenuated this shifting or there is predominance of sulphomucin as in control group. (Figure 7).

### Effect of Llycyrrhizic Acid and DMH on Mast Cell Infiltration

**Figure 8:**
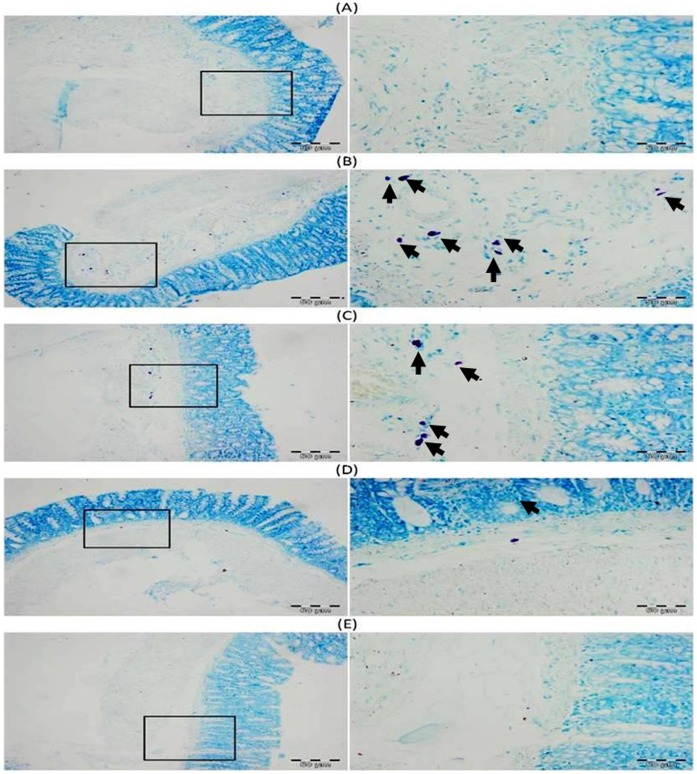
Photomicrographs depicting mast cells staining. In DMH treated group (Group II), there is infiltration of mast cells in the sub-mucosal layer below the lamina propria of the colonic section. Pretreatment with glycyrrhizic acid attenuated the infiltration of mast cells in Group III and Group IV as compared to Group II. There is no mast cells infiltration in colonic sections of Group I and Group V. Insets at the right panel show a magnified view (40x magnifications) of the insets showed at the left panel (10x magnifications).

In DMH treated group (Group II), there is infiltration of mast cells in the sub-mucosal layer below the lamina propria of the colonic section. Treatment with glycyrrhizic acid attenuated the infiltration of mast cells in Group III and IV as compared to Group II. There is no mast cells infiltration in colonic sections of Group I and Group V. (Figure 8).

### Effect of Glycyrrhizic Acid and DMH on the Expression of Ki-67, NF-kB-p65, COX-2, iNOS, VEGF, p53, Caspase-9 and Cleaved Caspase-3 in Colonic Tissue

**Figure 9:**
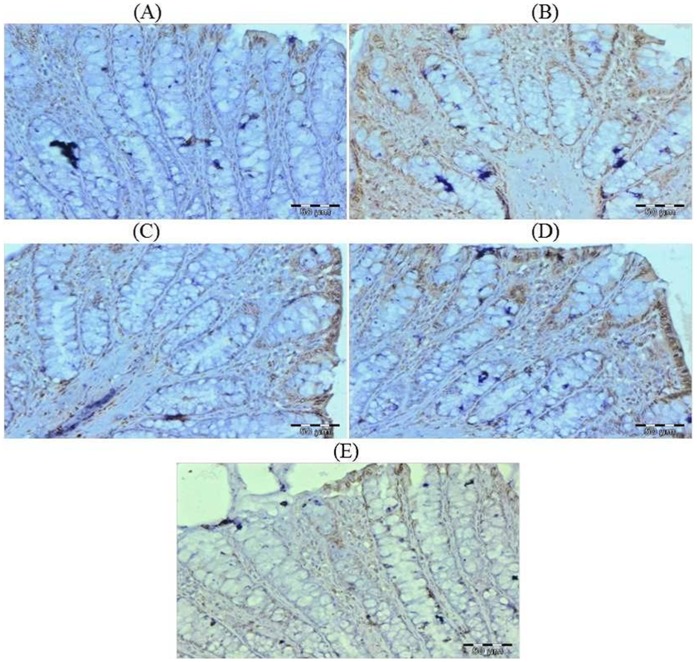
Photomicrographs depicting immunohistochemical staining of Ki-67. For immunohistochemical analyses, brown colour indicates specific immunostaining of Ki-67 and light blue colour indicates nuclear haematoxylin staining. The colonic section of DMH-treated group (Group II) has more Ki-67 immunopositive staining as indicated by brown colour as compared to control group (Group I) while treatment with glycyrrhizic acid in Group III and Group IV reduced Ki-67 immunostaining as compared to Group II. However there was no significant difference in the Ki-67 immunostaining in Group V as compared to Group I. Original magnification: 40x.

**Figure 10:**
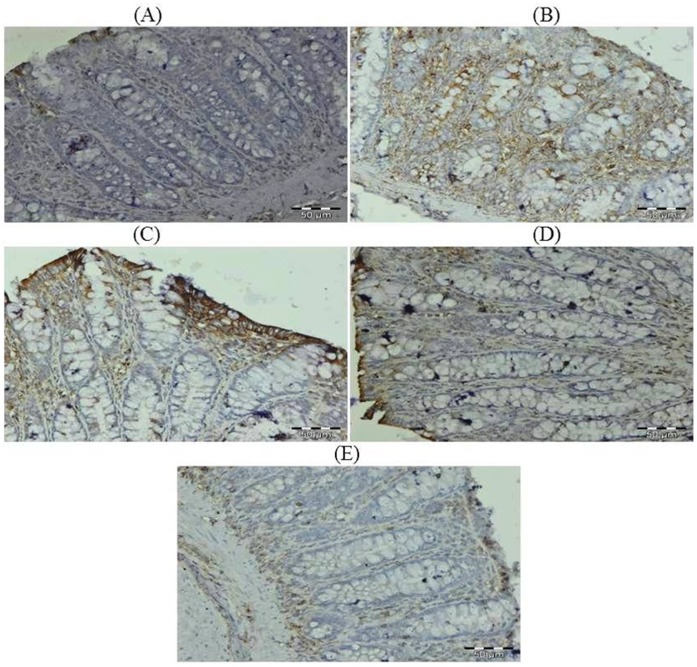
Photomicrographs depicting immunohistochemical staining of NF-kB. For immunohistochemical analyses, brown colour indicates specific immunostaining of NF-kB and light blue colour indicates nuclear haematoxylin staining. The colonic section of DMH-treated group (Group II) has more NF-kB immunopositive staining as indicated by brown colour as compared to control group (Group I) while treatment with glycyrrhizic acid in Group III and Group IV reduced NF-kB immunostaining as compared to Group II. However there was no significant difference in the NF-kB immunostaining in Group V as compared to Group I. Original magnification: 40x.

**Figure 11:**
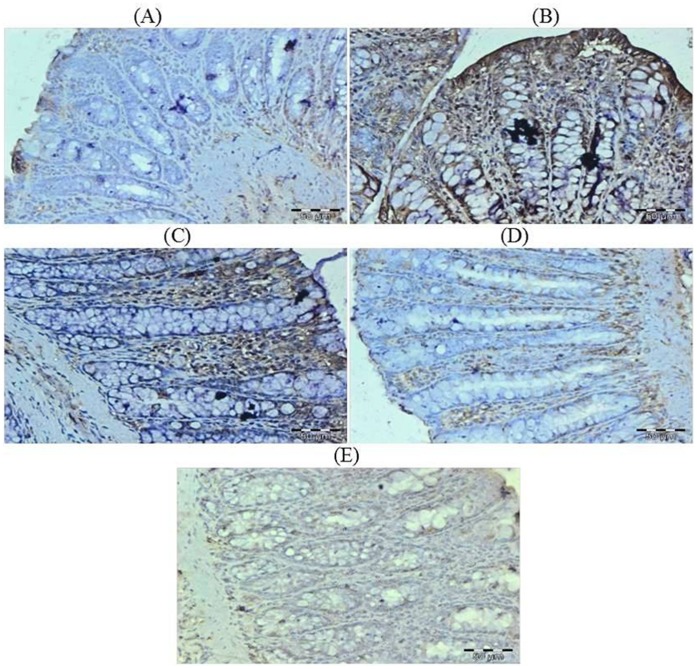
Photomicrographs depicting immunohistochemical staining of COX-2. For immunohistochemical analyses, brown colour indicates specific immunostaining of COX-2 and light blue colour indicates nuclear haematoxylin staining. The colonic section of DMH-treated group (Group II) has more COX-2 immunopositive staining as indicated by brown colour as compared to control group (Group I) while treatment with glycyrrhizic acid in Group III and Group IV reduced COX-2 immunostaining as compared to Group II. However there was no significant difference in the COX-2 immunostaining in Group V as compared to Group I. Original magnification: 40x.

**Figure 12:**
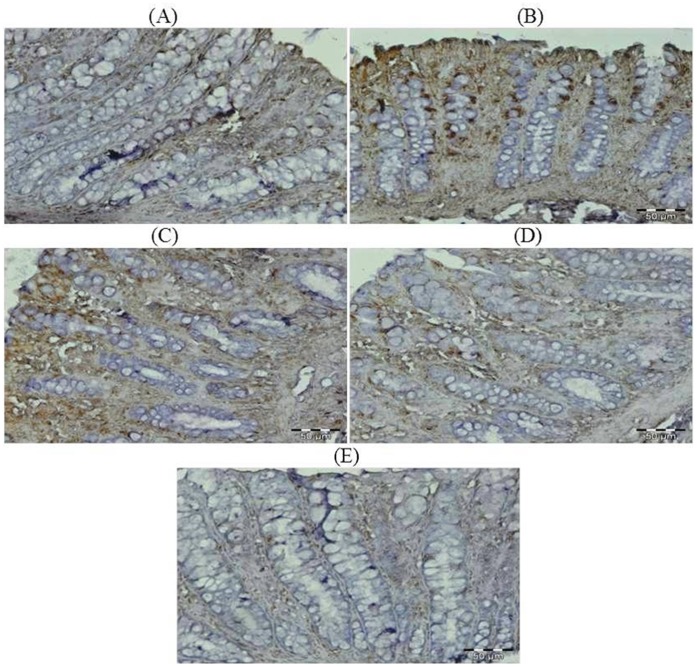
Photomicrographs depicting immunohistochemical staining of iNOS. For immunohistochemical analyses, brown colour indicates specific immunostaining of iNOS and light blue colour indicates nuclear haematoxylin staining. The colonic section of DMH-treated group (Group II) has more iNOS immunopositive staining as indicated by brown colour as compared to control group (Group I) while treatment with glycyrrhizic acid in Group III and Group IV reduced iNOS immunostaining as compared to Group II. However there was no significant difference in the iNOS immunostaining in Group V as compared to Group I. Original magnification: 40x.

**Figure 13:**
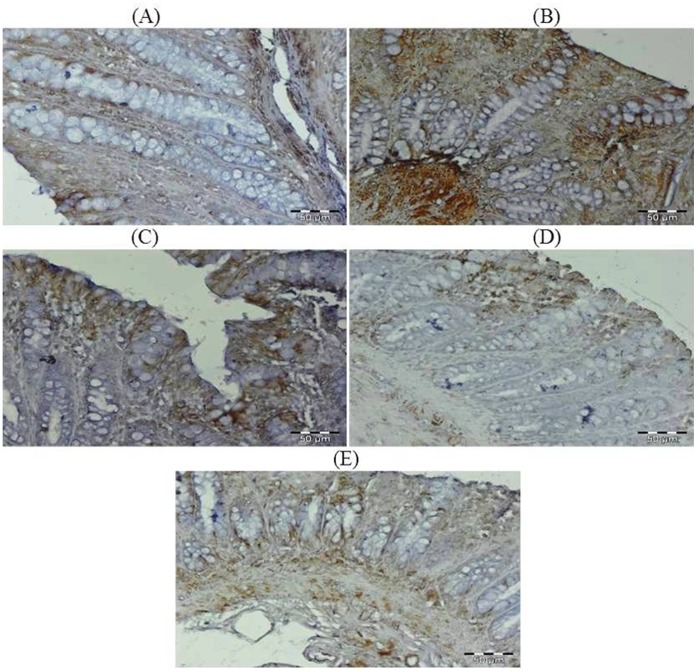
Photomicrographs depicting immunohistochemical staining of VEGF. For immunohistochemical analyses, brown colour indicates specific immunostaining of VEGF and light blue colour indicates nuclear haematoxylin staining. The colonic section of DMH-treated group (Group II) has more VEGF immunopositive staining as indicated by brown colour as compared to control group (Group I) while treatment with glycyrrhizic acid in Group III and Group IV reduced VEGF immunostaining as compared to Group II. However there was no significant difference in the VEGF immunostaining in Group V as compared to Group I. Original magnification: 40x.

**Figure 14:**
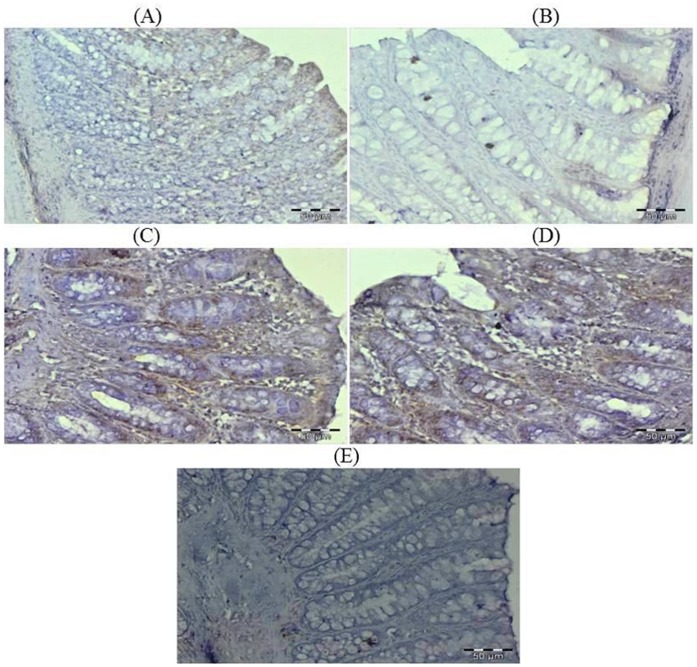
Photomicrographs depicting immunohistochemical staining of p53. For immunohistochemical analyses, brown colour indicates specific immunostaining of p53and light blue colour indicates nuclear haematoxylin staining. The colonic section of DMH-treated group (Group II) has reduced immunopositive staining of p53 as indicated by brown colour as compared to control group (Group I) while treatment of glycyrrhizic acid in Group III and Group IV enhanced the immunopositive staining of p53 as compared to Group II. However there was no significant difference in the p53 immunostaining in Group V as compared to Group I. Original magnification: 40x.

**Figure 15:**
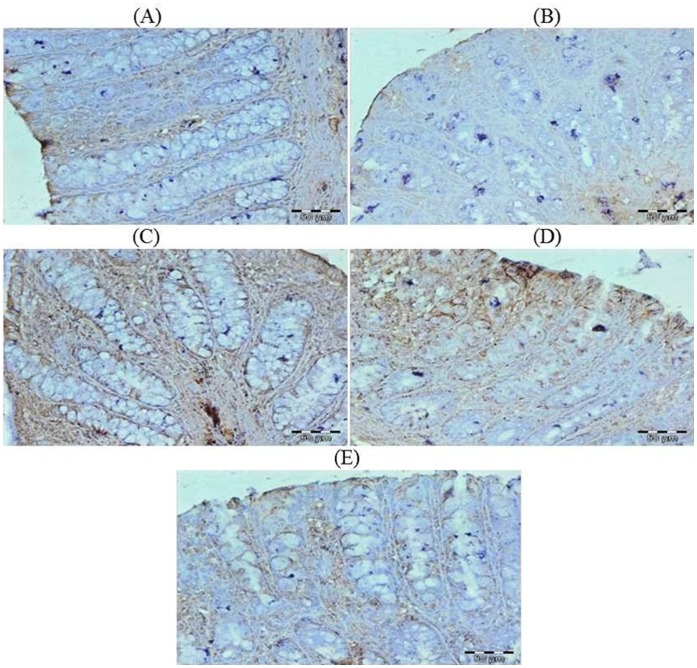
Photomicrographs depicting immunohistochemical staining of caspase-9. For immunohistochemical analyses, brown colour indicates specific immunostaining of caspase-9 and light blue colour indicates nuclear haematoxylin staining. The colonic section of DMH-treated group (Group II) has reduced immunopositive staining of caspase-9 as indicated by brown colour as compared to control group (Group I) while treatment of glycyrrhizic acid in Group III and Group IV enhanced the immunopositive staining of caspase-9 as compared to Group II. However there was no significant difference in the caspase-9 immunostaining in Group V as compared to Group I. Original magnification: 40x.

**Figure 16:**
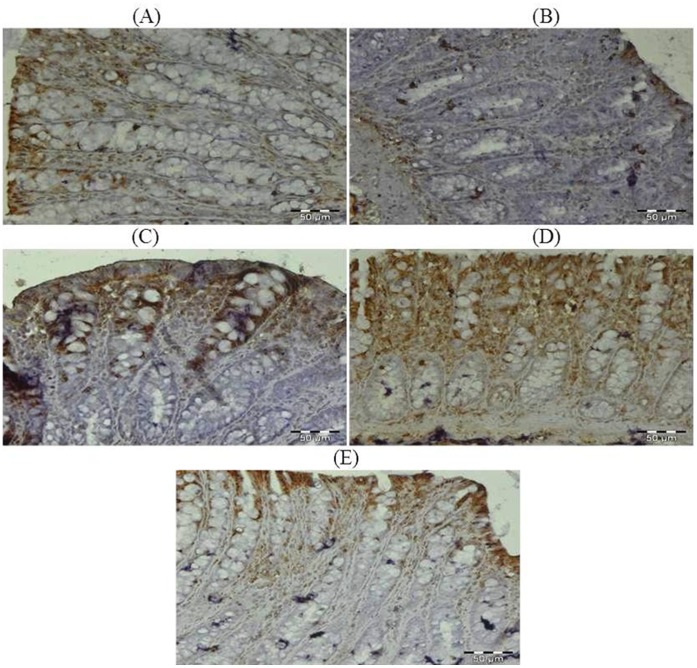
Photomicrographs depicting immunohistochemical staining of cleaved caspase-3. For immunohistochemical analyses, brown colour indicates specific immunostaining of cleaved caspase-3 and light blue colour indicates nuclear haematoxylin staining. The colonic section of DMH-treated group (Group II) has reduced immunopositive staining of cleaved caspase-3 as indicated by brown colour as compared to control group (Group I) while treatment of glycyrrhizic acid in Group III and Group IV enhanced the immunopositive staining of cleaved caspase-3 as compared to Group II. However there was no significant difference in the cleaved caspase-3 immunostaining in Group V as compared to Group I. Original magnification: 40x.

The colonic sections of DMH treated group (Group II) have more Ki-67, NF-kB, COX-2, iNOS and VEGF immunopositive staining while reduced p53, caspase-9 and cleaved caspase-3 immunopositive staining in DMH treated group (arrows) as indicated by brown colour as compared to control group (Group I). Treatment of glycyrrhizic acid reduced the immunostaining of Ki-67, NF-kB, COX-2, iNOS and VEGF while enhanced the immunostaining of p53, caspase-9 and cleaved caspase-3 in Group III and IV as compared to Group II. However, there were no significant differences in the immunostaining of all proteins in Group V as compared to Group I. For immunohistochemical analyses, brown colour indicates specific immunostaining of Ki-67, NF-kB, COX-2, iNOS, VEGF, p53, caspase-9 and cleaved caspase-3, and light blue colour indicates haematoxylin staining. Original magnification: 40x. (Figure 9, 10, 11, 12, 13, 14, 15, 16).

### Effect of Glycyrrhizic Acid and DMH on the Expression of Connexin-43 in Colonic Tissue

**Figure 17:**
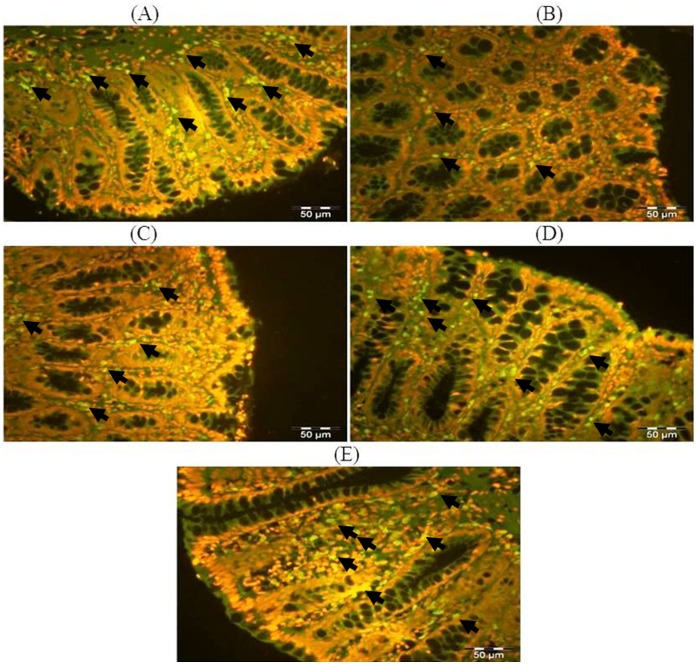
Photomicrographs depicting fluorescent immunohistochemical staining of connexin-43. For fluorescent immunohistochemical analyses, green colour indicates specific immunostaining of connexin-43 and red colour indicates nuclear propidium iodide staining. The colonic section of DMH treated group (Group II) has reduced connexin-43 immunopositive staining (arrows) as indicated by brown colour as compared to control group (Group I) while treatment with glycyrrhizic acid attenuated connexin-43 immunostaining in Group III and Group IV as compared to Group II. However, there was no significant difference in the connexin-43 immunostaining in Group V as compared to Group I. Original magnification: 40×.

The colonic sections of DMH treated group (Group II) have reduced expression of connexin-43 immunopositive staining (arrows) as indicated by green color as compared to control group (Group I) while treatment with glycyrrhizic acid in Group III and IV attenuated the immunostaining of connexin-43 as compared to Group II. However, there were no significant differences in the immunostaining of connexin-43 in Group V as compared to Group I. For fluorescent immunohistochemical analyses, green color indicates specific immunostaining of connexin-43, and red colour indicates propidium iodide staining. Original magnification: 40x. (Figure 17).

### Effect of Glycyrrhizic Acid and DMH on the Level of TNF-α in Colonic Tissue

**Figure 18:**
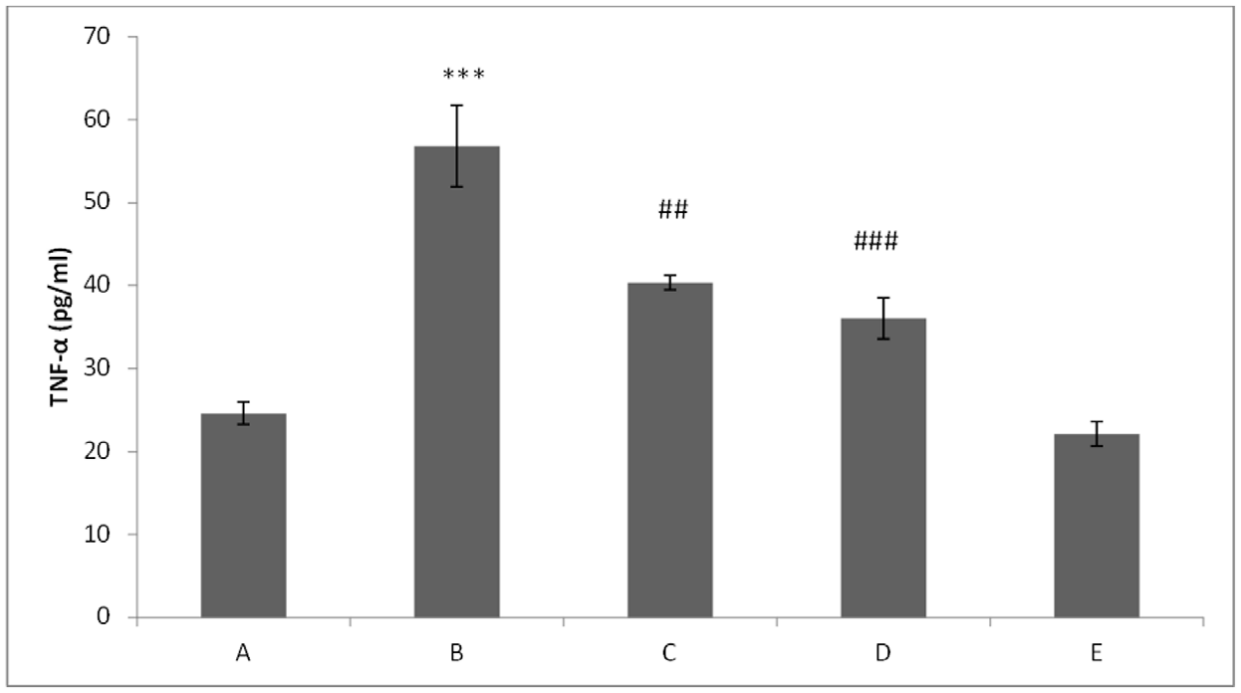
Effect of glycyrrhizic acid and DMH on TNF-α level. In DMH-treated group, the level of TNF-α was increased significantly (***p<0.001) as compared to control group. While treatment with glycyrrhizic acid significantly attenuated the level of TNF-α in Group III (##p<0.01) and Group IV (###p<0.001) as compared to Group II. There was no significant difference in the level of TNF-α in Group V as compared to Group I.

In DMH treated (Group II), the level of TNF-α was found to be significantly (p<0.001) elevated as compared to control group (Group I). Treatment with glycyrrhizic acid significantly attenuated the level of TNF-α in Group III (p<0.01) and IV (p<0.001) as compared to Group II. There is no significant difference between the levels of TNF-α in Group V as compared to Group I. (Figure 18).

### Effect of Glycyrrhizic Acid and DMH on the Colonic Histoarchitecture

**Figure 19:**
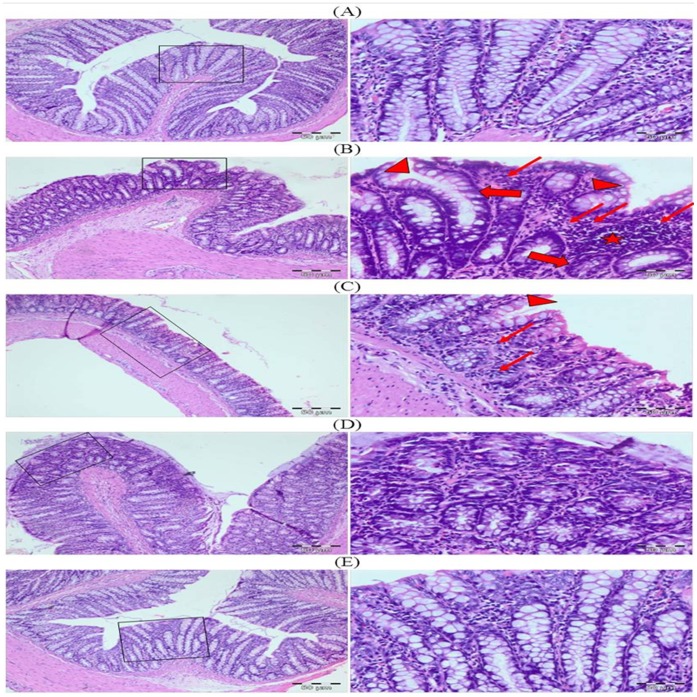
Photomicrographs depicting histology of rat colon. The histological sections of control group showed normal histoarchitecture while DMH treated group (Group II) exhibited intense inflammatory cells infiltration, irregular glandular structure along with crypt ablation. In Group III and Group IV, histological sections showed that treatment with glycyrrhizic acid showed protection against DMH induced colonic damage. Colonic sections of Group V (only glycyrrhizic acid treated group) displayed normal histology as similar to that of Group I (control group). Insets at the right panel show a magnified view (40x magnifications) of the insets showed at the left panel (10× magnifications).

The H&E stained sections of control group showed normal histoarchitecture with mild inflammatory cells infiltration while DMH treated group (Group II) exhibited intense inflammatory cells infiltration, irregular glandular structure along with crypt ablation. It has been observed that treatment with glycyrrhizic acid showed protection against DMH induced mucosal damage with marked reduction in the inflammatory cells infiltration in group III and IV. Colonic sections of Group V (only glycyrrhizic acid treated group) displayed normal histology as similar to that of Group I (control group). (Figure 19).

## Discussion

To the best of our knowledge, this is the first report to demonstrate the potential of glycyrrhizic acid to suppress DMH-induced ACF and MDF. In this study, we have observed that treatment with glycyrrhizic acid suppresses the development of early markers of colon cancer i.e., ACF and MDF. It has been reported that glycyrrhizic acid has chemopreventive effects against colon carcinogenesis in Apc^+/min^ mouse model and UV-B radiation induced skin tumorigenesis in SKH-1 hairless mouse model [39], [54].

Glycyrrhizic acid supplementation reduced the number of ACF but not at the significant level in both the group i.e., initiation and post-initiation group as compared to DMH treated group. The number of MDF is reduced significantly in both the group i.e., initiation and post-initiation group as compared to DMH treated group. These findings indicate that glycyrrhizic acid effectively suppresses one of the early events of colon carcinogenesis in Wistar rats.

Mucins are high molecular weight, heavily glycosylated proteins secreted by epithelial cells of the colon which forms a protective mucous layer in the form of gel in intestinal lumen [55], [56]. The loss of the mucous layer might induce inflammation in this region since this region is now more exposed to various noxious agents present in colonic lumen. Our results showed that there was marked mucin depletion (blue color) in DMH treated group as compared to control group while supplementation with glycyrrhizic acid attenuated the mucin depletion to some extent.

There are basically two types of mucin i.e., sulphomucin (brown color) and sialomucin (blue color). The normal human colorectal mucosa and the distal part of the rat colon predominantly secrete sulphomucin. Previous studies carried out in histological sections have shown that apparently normal colonic mucosa from patients with colon cancer and dysplastic foci observed in the distal colon of carcinogen-treated rats produce predominantly sialomucins instead of sulphomucins [57]–[60]. We observed only the distal part of the rat colons since the distal colon shows a pattern of mucus production similar to that of the normal human colorectal mucosa in which sulphomucin secretion predominates and since precancerous alterations in the human and distal rat colon are accompanied by a shift from sulphomucin to sialomucin secretion [57], [58]. In this study, there is predominance of sialomucin (blue color) in the colon of DMH-treated rats as compared to control rats which exhibited predominance of sulphomucin (brown color) while treatment with glycyrrhizic acid attenuated this shifting from sulphomucin to sialomucin.

Previous investigations have been shown that glycyrrhizic acid has anti-inflammatory property [61]–[63]. Infiltration of mast cells is the sign of initiation of inflammation. Recent experiments indicate that mast cell infiltration can enhance carcinogenesis [64]–[68] and they have also long been known to drive angiogenesis and tumour growth [67]. In this study, it was observed that there is marked infiltration of mast cells in the sub-mucosal layer in DMH-treated group while there is no mast cells infiltration in control group. Treatment with glycyrrhizic acid markedly reduced the infiltration of mast cells within the sub-mucosal layer which clearly indicates the anti-angiogenic potential of glycyrrhizic acid.

**Figure 20:**
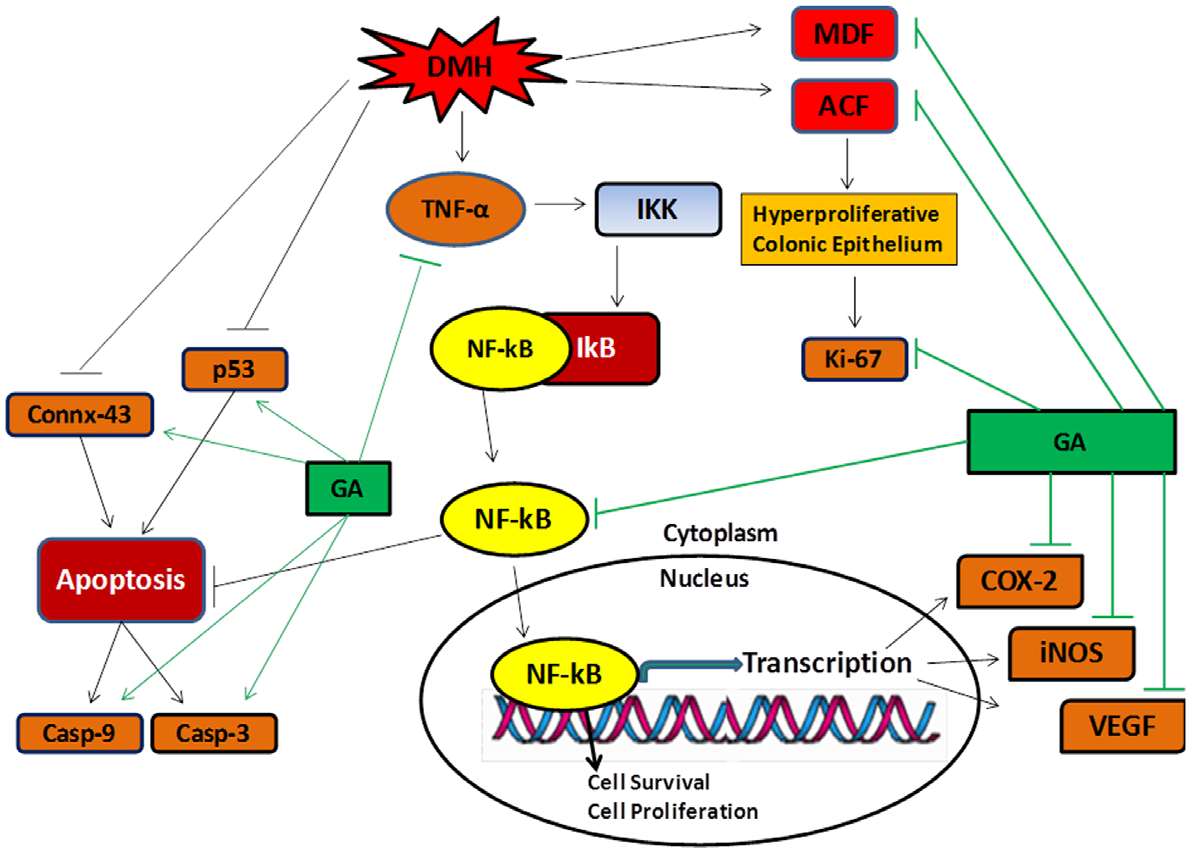
Targets of action of glycyrrhizic acid (GA) against DMH induced preneoplastic lesions and damages in colon of Wistar rats. GA modulates DMH induced ACF and MDF in colon of Wistar rats. DMH treatment causes inflammation which induces the enhance expression of inflammatory markers like TNF-α, NF-kB, COX-2, iNOS along with increased infiltration of mast cells. GA supplementation attenuates the expression of TNF-α, NF-kB, COX-2, and iNOS and reduces the infiltration of mast cells within the submucosal layer of the colon. DMH treatment reduces the expression of connexin-43 and p53 and GA supplementation attenuates the expression of connexin-43 and p53. The activated NF-kB leads to cell survival which in turn reduces the expression of apoptotic markers like caspase-9 and cleaved caspase-3 and treatment with glycyrrhizic acid attenuates the expression of caspase-9 and cleaved caspase-3. GA also attenuates the DMH-induced enhanced expression of Ki-67 and VEGF which is a marker of cell proliferation and angiogenesis respectively. Casp-9 = Caspapse-9; Casp-3 = Cleaved Caspase-3; DMH = 1,2-dimethylhydrazine; TNF-α = Tumor Necrosis Factor-alpha, NF-kB = Nuclear Factor-kappa B; IKK = I kappa B kinase; IkB = I kappa B; Conn-43 = Connexin-43; COX-2 = Cycloxygenase-2; iNOS = inducible Nitric Oxide Synthase; VEGF = Vascular Endothelial Growth Factor; GA = Glycyrrhizic acid; ACF = Aberrant Crypt Foci; MDF = Mucin Depleted Foci.

NF-kB activated in response to inflammation [30] and carcinogens [68]. Once activated, NF-kB transactivate the genes encoding TNF-α, COX-2, iNOS, and VEGF. Our findings showed that there is enhanced immunopositive staining of NF-kB, COX-2, iNOS and VEGF, and significantly elevated level of TNF-α in DMH-treated group as compared to control group. Treatment with glycyrrhizic acid markedly attenuated the immunopositive staining of NF-kB, COX-2, iNOS and VEGF, and also significantly attenuated the level of TNF-α. These results suggest that glycyrrhizic acid has anti-inflammatory and anti-angiogenic potential. (Figure 20).

In the previous studies, it has been observed that glycyrrhizic acid suppresses the hyperproliferative responses via down-regulation of the expression of proliferation marker i.e., proliferating cell nuclear antigen (PCNA) [54]. Ki-67 is also a cell proliferation marker which is a nuclear protein mainly express in proliferating cells [69]. In our study, it was observed that DMH-treated group has more Ki-67 immunopositive staining as compared to control group while treatment with glycyrrhizic acid significantly attenuated the Ki-67 immunopositive staining. These results further corroborated with the previous findings that glycyrrhizic acid suppresses the hyperproliferative responses in the colon of Wistar rats. (Figure 20).

Connexin-43 is one of the transmembrane protein that form channels between adjacent cells known as gap junctions [70]. Connexin-43 also acts as a colorectal cancer tumor suppressor and the expression of connexin-43 is commonly down-regulated in tumors, leading to loss of gap junctional intercellular communication [71]. It has also been reported to regulate growth of colon cancer cells via inducing apoptosis [71]. The p53 is a tumor suppressor protein and also acts as a transcription factor that regulates the transcription of genes involved in cell cycle, DNA repair and apoptosis [72]. p53 induces apoptosis via intrinsic pathway (mitochondrial pathway) by evoking cytochrome c release from the mitochondria that leads to the activation of Apaf-1 and caspase 9. Caspase-9 in turn ultimately leads to the activation of caspase-3 [73].

In this study, it was observed that DMH-treated group has reduced immunopositive staining of connexin-43 as well as p53 as compared to control group which showed that there is reduced expression of connexin-43 and p53 while treatment with glycyrrhizic acid significantly attenuated the immunopositive staining of connexin-43 and p53. It was also observed that DMH-treated group has reduced immunopositive staining of caspase-9 and cleaved caspase-3 as compared to control group while treatment with glycyrrhizic acid significantly enhanced the immunopositive staining of caspase-9 and cleaved caspase-3. These results exhibited that treatment with glycyrrhizic acid induce apoptosis and these results corroborated with the previous reports which showed that glycyrrhizic acid induces apoptosis in several cancer cell lines [45]–[47]. (Figure 20).

Histological findings revealed that control group showed normal histoarchitecture with mild infiltration of inflammatory cells as well as intact mucosal glandular structure while DMH-treated group exhibited massive infiltration of inflammatory cells in the lamina propria, distorted mucosal glandular architecture along with crypt ablation and crypt abscess formation. Treatment with glycyrrhizic acid strongly suppressed the infiltration of inflammatory cells in the mucosal layer, reduced the severity of submucosal edema, crypt abscess formation and crypt ablation induced by DMH in the colon of Wistar rats. Histological findings clearly revealed that glycyrrhizic acid has strong anti-inflammatory property. The above mentioned findings corroborated with the histological data which exhibited the protective effects of glycyrrhizic acid against DMH-induced colonic damage.

Overall the aforementioned findings of the study accepts the hypothesis but with some limitation of the use of glycyrrhizic acid. Chronic excessive ingestion of glycyrrhizic acid has been reported to induce hypokalemia and high blood pressure in a subset of people. Therefore, it is expected that if equivalent doses of glycyrrhizic acid which we have used in our study, were used in humans, may develop hypertension and/or Hypokalemia [39].

It can be concluded from the findings of the present study that glycyrrhizic acid has chemopreventive potential against DMH-induced colon carcinogenesis via suppressing the development of precancerous lesions i.e., ACF and MDF in the colon of Wistar rats. The precise mechanism of protective action of glycyrrhizic acid against DMH induced colonic damage is still unknown but the probable mechanism may be through the attenuation of hyperproliferation, inflammation, angiogenesis and apoptotic responses in the colon of Wistar rats. Further studies are warranted to elucidate the exact protective mechanism of glycyrrhizic acid.
